# Effect of opioid-free anesthesia on postoperative nausea and vomiting after gynecological surgery: a systematic review and meta-analysis

**DOI:** 10.3389/fphar.2023.1330250

**Published:** 2024-01-04

**Authors:** Zheng Zhang, Chengwei Li, Lin Xu, Xinyi Sun, Xiaojie Lin, Penghui Wei, Jianjun Li

**Affiliations:** ^1^ Department of Anesthesiology, Qilu Hospital of Shandong University, Jinan, China; ^2^ Department of Anesthesiology, Qilu Hospital (Qingdao), Cheeloo College of Medicine, Shandong University, Qingdao, China

**Keywords:** opioid-free anesthesia, postoperative nausea and vomiting, gynecological surgery, quality of recovery, pain

## Abstract

**Background:** Postoperative nausea and vomiting (PONV) is a common complication, that can reduce patient satisfaction and may lead to serious consequences, such as wound dehiscence. Many strategies have been proposed to prevent PONV; however, it remains common, especially in high-risk surgeries such as gynecological surgery. In recent years, opioid-free anesthesia has been widely studied because it minimizes adverse reactions of opioids, such as nausea, vomiting, and itching; however, conclusions have been inconsistent. Therefore, we conducted this meta-analysis to investigate the effects of opioid-free anesthesia on PONV in patients undergoing gynecological surgery.

**Methods:** A systematic search of the PubMed, Web of Science, Cochrane Library, and Embase databases, from inception to 28 August 2023, was performed. Keywords and other free terms were used with Boolean operators (OR and, AND) to combine searches. This review was performed in accordance with Preferred Reporting Items for Systematic Reviews and Meta-Analyses (PRISMA).

**Results:** Six studies involving 514 patients who underwent gynecological surgery were included. The forest plot revealed that the incidence of PONV (risk ratio = 0.52; *p* < 0.00001) and consumption of postoperative antiemetics use (risk ratio = 0.64; *p* = 0.03) were significantly lower in the opioid-free anesthesia group. In addition, opioid-free anesthesia improved the quality of recovery (mean difference = 4.69; *p* < 0.0001). However, there were no significant differences in postoperative pain scores (mean difference = 0.05; *p* = 0.85), analgesic use (risk ratio = 1.09; *p* = 0.65), and the time of extubation (mean difference = −0.89; *p* = 0.09) between the opioid-free anesthesia and control groups.

**Conclusion:** OFA reduces PONV and the use of antiemetic drugs. In addition, it improves the quality of postoperative recovery. However, OFA can not reduce the postoperative pain scores, analgesic use and the time of extubation. Due to the strength of the evidence, we cannot support OFA as an ideal anesthesia method in gynecological surgery, and the implementation of anesthesia strategies should be case-by-case.

Systematic Review Registration: [https://www.crd.york.ac.uk/prospero/display_record.php?RecordID=462044], identifier [CRD42023462044]

## Introduction

As an important part of general anesthesia, opioids exert a strong intraoperative anti-nociceptive stimulus and analgesic effect, and ensure stable vital signs in patients undergoing surgical procedures (Shinoda et al., 2013; Adams et al., 2023). However, improper use of opioids can also cause many adverse reactions such as postoperative nausea and vomiting (PONV), hyperalgesia, respiratory depression, and inflammatory responses, which prolong the length of postoperative hospital stay and increases medical costs (Macintyre et al., 2022). Moreover, opioid use for postoperative analgesia can lead to opioid addiction and abuse (Vadivelu et al., 2018). The incidence of PONV in patients undergoing gynecological surgery involving opioids is 50%–80%, and patients often experience postoperative hypoxemia and bradycardia due to opioid residue (Madej and Simpson, 1986; Zorrilla-Vaca et al., 2022).

With the emerging concept of enhanced recovery after surgery, opioid-free anesthesia (OFA) has been gradually introduced in an increasing number of surgeries (Moningi et al., 2019). OFA is a multimodal anesthesia method, which combines multiple drugs or methods such as sedatives, N-methyl-D-aspartate receptor antagonists, local anesthetics, anti-inflammatory drugs and α2 receptor agonists to enhance intraoperative analgesia and minimize the use of opioids during the perioperative period. Elkassabany et al. defines OFA as a perioperative treatment strategy from admission to discharge, that is, to perform anesthesia and analgesia in a non-opioid mode as far as possible, and reserve opioids for pain that cannot be relieved by other methods (Elkassabany and Mariano, 2019). Forget et al. believes that OFA can be defined as a combination of different opioid-sparing techniques to achieve opioid-free anesthesia (Forget, 2021). Mulier et al. distinguishes OFA from opioid-free analgesia, and believes that OFA means that opioids are not used before or during surgery until the patient is awake (Mulier and Dekock, 2017). It can reduce the risk for common opioid-related adverse reactions, such as postoperative respiratory depression, and PONV, and reduce the potential for dependence and addiction of patients to opioids (Feenstra et al., 2023). OFA has been widely used in bariatric and thoracic surgeries, and ideal results have been obtained (Selim et al., 2022; Mieszczański et al., 2023).

We conducted this systematic review and meta-analysis to compare and summarize postoperative outcomes, including PONV, antiemetic use, pain scores, and analgesic use, time of extubation and QoR-40 score between OFA and opioid-based anesthesia in gynecological surgery.

## Methods

This review was performed by the Preferred Reporting Items for Systematic Reviews and Meta-Analyses (PRISMA) guidelines for systematic review (Page et al., 2021). This systematic review and meta-analysis were included in PROSPERO (registration number: CRD42023462044).

### Search strategy

The PubMed, Web of Science, Cochrane Library, and Embase databases were systematically searched from inception to 28 August 2023, for relevant studies published in English. The search terms used were as follows: ((opioid free [Title/Abstract]) OR (opioid-free [Title/Abstract])) AND ((“Gynecologic Surgical Procedures" [Mesh]) OR (((((((((((((((((((Procedures, Gynecologic Surgical [Title/Abstract]) OR (Surgical Procedure, Gynecologic [Title/Abstract])) OR (Surgery, Gynecological [Title/Abstract])) OR (Gynecological Surgeries [Title/Abstract])) OR (Gynecological Surgery [Title/Abstract])) OR (Surgeries, Gynecological [Title/Abstract])) OR (Gynecologic Surgical Procedure [Title/Abstract])) OR (Surgical Procedures, Gynecologic [Title/Abstract])) OR (Gynecological Surgical Procedure [Title/Abstract])) OR (Gynecological Surgical Procedures [Title/Abstract])) OR (Procedure, Gynecological Surgical [Title/Abstract])) OR (Procedures, Gynecological Surgical [Title/Abstract])) OR (Surgical Procedure, Gynecological [Title/Abstract])) OR (Surgical Procedures, Gynecological [Title/Abstract])) OR (Procedure, Gynecologic Surgical [Title/Abstract])) OR (Gynecologic Surgery [Title/Abstract])) OR (Gynecologic Surgeries [Title/Abstract])) OR (Surgeries, Gynecologic [Title/Abstract])) OR (Surgery, Gynecologic [Title/Abstract])))

All retrieved citations were downloaded and imported into a reference management tool (EndNote, Clarivate, London, United Kingdom) (Bramer et al., 2017). Duplicate studies were eliminated, titles and abstracts were reviewed to exclude studies that did not meet the inclusion criteria, and the remaining articles were analyzed and further screened according to the inclusion and exclusion criteria.

### Selection criteria

The inclusion criteria were developed based on the “PICOS” principle: Patients, those underwent gynecological surgery or examination; Intervention, opioid-free anesthesia; Comparison, opioid-based anesthesia; Outcomes, incidence of the opioid-free anesthesia group and the control group; and Study design, randomized controlled trial.

Studies with no available or incomplete data, meta-analysis, systematic reviews, or retrospective studies, and those addressing pediatric or emergency surgery were excluded.

### Data collection and assessment of risk of bias

Two researchers independently extracted and collected the following information: ZZ extracted the title; name of first author; year of publication; type of surgery; number of patients in both groups; CW L collected the characteristics of the patients, and the required outcomes in the two groups. The data extraction author was blinded.

ZZ used the Cochrane Risk of Bias 2.0 to assess the quality of the included studies (Higgins et al., 2011). CW L used the Begg test and Egger test in Stata 16.0 software to analyze publication bias. Any disagreement was resolved through discussion with another author.

### Data analysis

Data analysis was performed using Review Manager 5.4 (Copenhagen: Nordic Cochrane Centre, The Cochrane Collaboration, 2020). Pooled risk ratio (RR) and 95% confidence interval (CI) for dichotomous results were calculated using the Mantel-Haenszel method. For continuous outcomes, mean difference (MD) and 95% CI were calculated using inverse-variance method. If only the median and interquartile range (IQR) were available, the Wan methods was used to converted data expressed as medians (interquartile range) to means ± standard deviations (Wan et al., 2014). The statistical heterogeneity was assessed using the chi-square test, and the I^2^ statistic was calculated. I^2^ values of approximately 25%, 50%, and 75% were considered low, moderate, and severe heterogeneity, respectively (I^2^ > 50% was used as the threshold to indicate significant heterogeneity in individual studies). Data analysis of the collated data was performed using a fixed-effects model when I^2^ ≤ 50, and a randomized-effects model when I^2^ > 50. *p* < 0.05 was considered statistically significant. Subgroup analysis of primary outcomes was performed by Stata 16.0 and divided into groups according to type of intraoperative opioid use (remifentanil vs sufentanil, fentanyl), methods used for maintenance of opioid-free anesthesia (volatile anesthesia combined with propofol vs propofol vs epidural anesthesia) in opioid-free anesthesia, methods of implementation of opioid-free anesthesia (epidural vs general anesthesia), and whether dexmedetomidine is used in opioids anesthesia (yes vs no). Sensitivity analysis was used for secondary outcomes with high heterogeneity and it was performed by removing one study at a time to estimate the effect of an individual study on the pooled results**.**

## Results

### Literature search and screening

**FIGURE 1:**
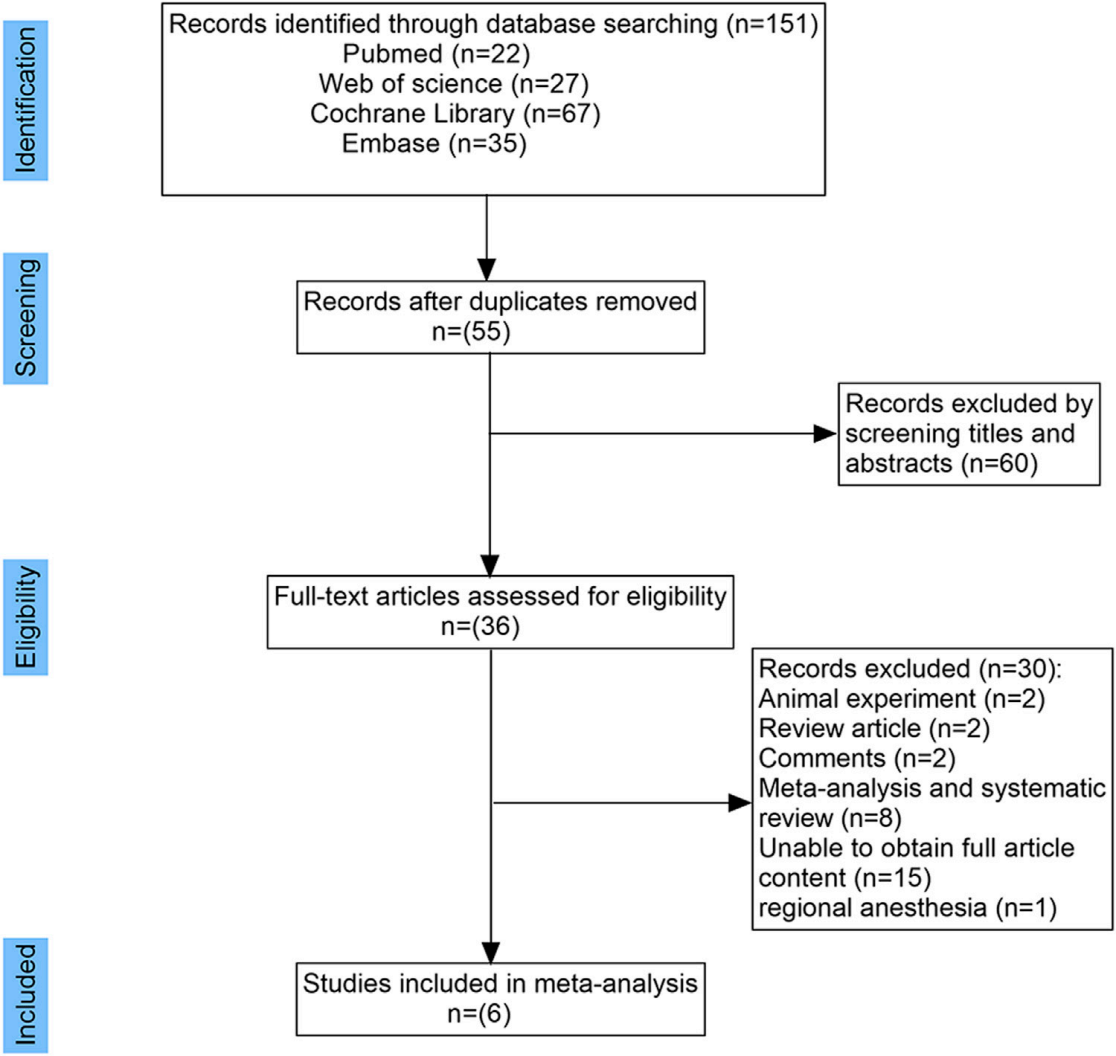
PRISMA flow diagram of study selection for the meta-analysis.

A total of 151 relevant studies were retrieved, and 115 were excluded. After reviewing the remaining 36 studies, 30 were excluded for reasons shown in Figure 1. Six studies involving 514 adult patients undergoing gynecological surgery or examination were included in this meta-analysis (Callesen et al., 1999; Hakim and Wahba, 2019; Massoth et al., 2021; Chen et al., 2022; Choi et al., 2022; Cha et al., 2023). Of the 6 included studies, 2 reported the use of fentanyl in the control group (Callesen et al., 1999; Hakim and Wahba, 2019), 3 reported the use of sufentanil (Massoth et al., 2021; Chen et al., 2022; Cha et al., 2023), and 1 reported the use of remifentanil in the control group (Choi et al., 2022). However, the OFA groups reported in the six studies used different regimens. The characteristics of the included studies are presented in Table 1.

**TABLE 1:** Characteristics of studies (n = 6).

Study	Type of surgery	Intraoperative regimen in opioid-free anesthesia group	Intraoperative regimen in control group	Number of patients in opioid-free anesthesia group	Number of patients in control group	Outcomes
Chen, 2023	Laparoscopic hysterectomy	Esketamine (0.3–0.5 mg/kg i.v. and 0.3 mg/kg/h for infusion)	Sufentanil (0.2–0.4 μg/kg i.v.)	39	38	-AUC of VAS.
		Dexmedetomidine (0.5 μg/kg i.v. and 0.1–0.3 μg/kg/min for infusion)	Remifentanil (8–10 μg/kg/h)			-Number of postoperative rescue analgesia required
		TAP block (15 mL of 0.25% ropivacaine)	TAP block (15 mL of 0.25% ropivacaine)			-PONV.
		Furbiprofen axate (50 mg)	Furbiprofen axate (50 mg)			-PSQI.
		Azasetron (10 mg)	Azasetron (10 mg)			-Intraoperative hemodynamic variables
		Propofol (2–2.5 mg/kg i.v. and 5–7 mg/kg/h for infusion)	Propofol (2–2.5 mg/kg i.v. and 5–7 mg/kg/h for infusion)			-Awakening and orientation recovery times
Cha, 2023	Hysteroscopy	Lidocaine (1.5 mg/kg i.v. and 1.5 mg/kg/h for infusion)	Sufentanil (0.3 μg/kg)	45	45	-QoR-40
		Sevoflurane (2%–3%)	Sevoflurane (2%–3%)			-Extubation time
		Propofol (2.0 mg/kg)	Propofol (2.0 mg/kg)			-Severe complications
						-Severe complications
Choi, 2022	Hysterectomya	Lidocaine (1.5 mg/kg i.v. and 1.5 mg/kg/h for infusion)	Remifentanil (3.5 ng/mL)	37	38	-QoR-40
		Dexmedetomidine (0.7 μg/kg i.v. and 0.5 μg/kg/h for infusion)	Desflurane (4%–6%)			-Postoperative pain score
		Desflurane (4%–6%)	Dexamethasone (5 mg)			-Intraoperative adverse events
	Myomectomyb	Dexamethasone (5 mg)	Palonosetron (75 μg)			-Stress hormones levels
		Palonosetron (75 μg)	Acetaminophen (1 g)			-Cystectomy enucleation events
		Acetaminophen(1 g)	Ketorolac (30 mg)			
	Adnexectomy	Ketorolac(30 mg)	Propofol (1.5–2 mg/kg)			-Postoperative adverse
		Propofol (1.5–2 mg/kg)				
Massoth, 2021	Hysterectomy	Esketamine (0.15 mg/kg i.v. and 0.15 mg/kg/h for infusion)	Sufentanil (0.3 μg/kg and 0.15 μg/kg)	76	76	-PONV.
	Endometriosis	Dexmedetomidine (0.6 μg/kg i.v. and 0.3 μg/kg/h for infusion)	Sevoflurane (MAC 0.8–1.0)			-Pain score
	Adnexectomy	Sevoflurane (MAC 1.0–1.4)	Dexamethasone (4 mg)			-Recovery characteristics
	Diagnostic laparoscopy	Dexamethasone (4 mg)	Ondansetron (4 mg)			
	Myoma enucleation	Ondansetron (4 mg)	Propofol (1–2 mg/kg)			-Morphine consumption
	Other	Propofol (1–2 mg/kg)				
Hakim, 2019	Laparoscopic gynecological	Dexmedetomidine (0.6 μg/kg i.v. and 0.2 μg/kg/h for infusion)	Fentanyl (1 μg/kg i.v. and 0.5 μg/kg/h for infusion)	40	40	-QoR-40
						-NRS.
						-PONV.
		Propofol (2 μg/kg i.v. and 5–10 mg/kg/h for infusion)	Propofol (2 μg/kg i.v. and 5–10 mg/kg/h for infusion)			-Time to first rescue analgesia
						-Number of rescue tramadol analgesia
Callesen, 1999	Abdominal hysterectomy	Bupivacaine (6 mL/h for bolus and 8 mL/h for infusion)	Fentanyl (3 μg/kg)	20	20	-Nausea
		Fascial injection (bupivacaine 15 mL)	Thiopental(4 mg/kg)			-Vomiting
			Morphine(2 mg)			-Pain score
		Tenoxicam (40 mg)	Bupivacaine (0.25% 8 mL)			-Bowel function score
			Isoflurane			

TAP, transversus abdominis plane; AUC, the area under the curve; VAS, visual analogue scale; PONV, postoperative nausea and vomiting; PSQI, pittsburgh sleep quality index; QoR-40, Quality of Recovery-40 questionnaire; OFA, opioid-free anesthesia; NRS, numeric rating scale.

### Quality assessment

**FIGURE 2:**
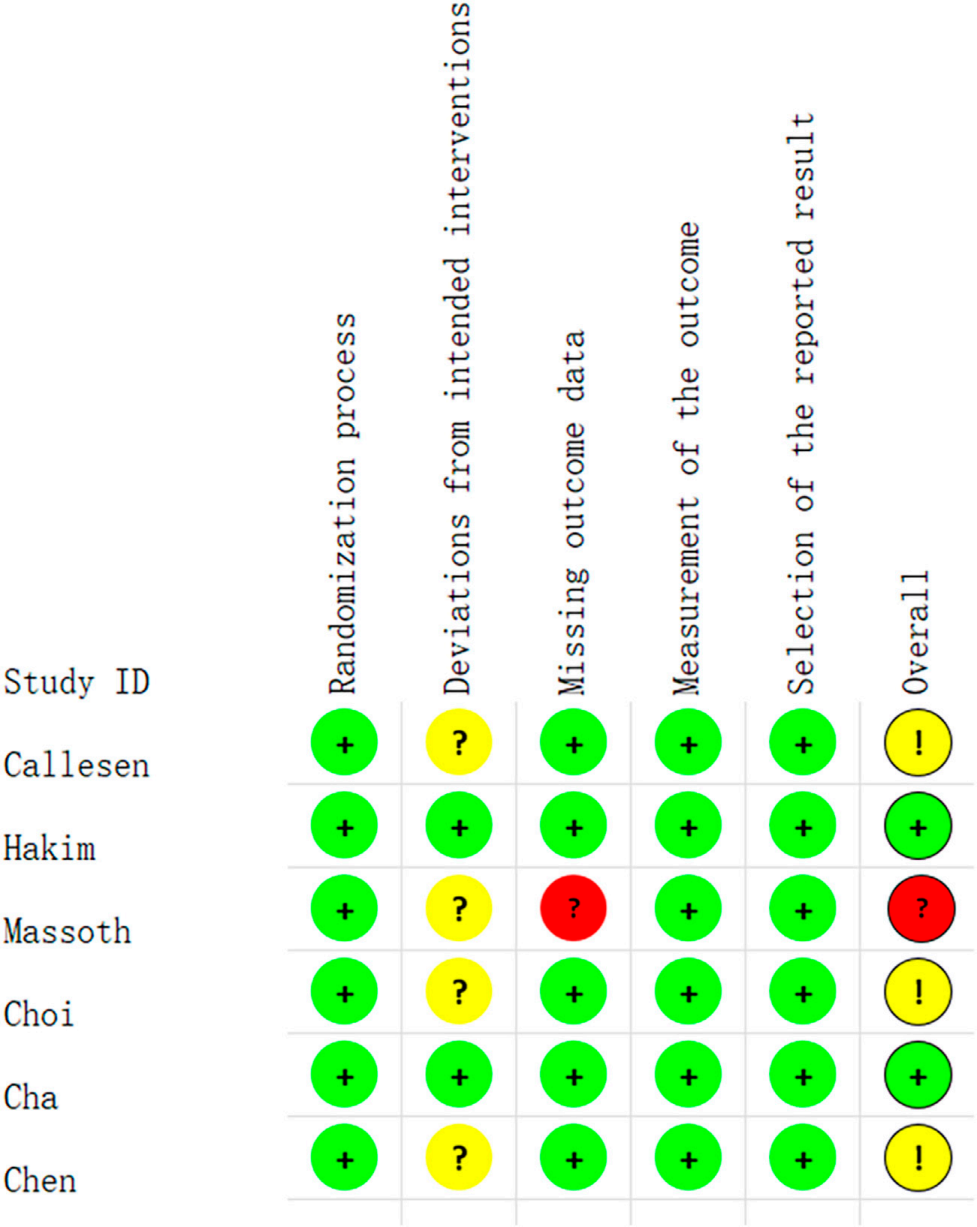
Graph of risks of bias of each included study.

Of the 6 studies, 2 studies were considered to have a low risk of bias (Hakim and Wahba, 2019; Cha et al., 2023). The study by Massoth et al. was considered likely to have a high attrition bias (Massoth et al., 2021). In addition, three studies were considered to have an unclear risk of bias (Callesen et al., 1999; Chen et al., 2022; Choi et al., 2022). The quality evaluation of the included studies was shown in Figure 2.

### Publication bias

According to the principle of publication bias, we counted the research reports with PONV (Callesen et al., 1999; Hakim and Wahba, 2019; Massoth et al., 2021; Chen et al., 2022; Choi et al., 2022; Cha et al., 2023) as the outcome (Begg’s *p* = 0.707, Egger’s *p* = 0.560), suggesting that the articles included in the research report had no publication bias. The result was shown in Table 2.

**TABLE 2:** Evaluation of publication bias.

Index	RR (95%CI)	Z	*p*-value	I^2^ (%)	I^2^’s P	Egger’s P	Begg’s P
PONV	0.52 [0.40, 0.66]	5.19	*p*< 0.00001	12	0.34	0.560	0.707

PONV, postoperative nausea and vomiting; RR, risk ratio; 95%CI, 95% confidence interval.

### PONV and rescue antiemetics

**FIGURE 3:**
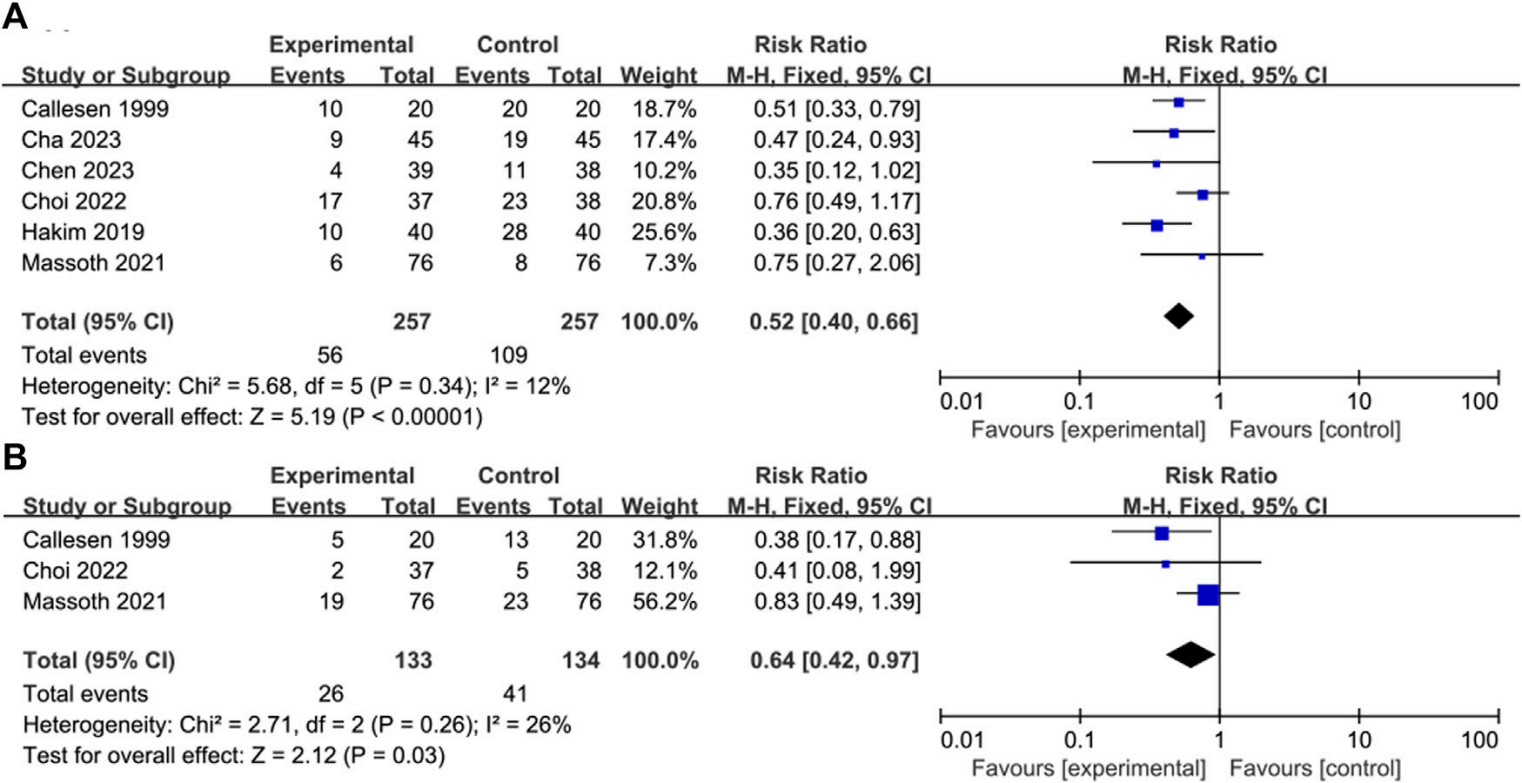
Forest plot of the **(A)** incidence of postoperative nausea and/or vomiting (PONV) **(B)** rescue antiemetics: opioid-free anesthesia vs control.

Six studies including 514 patients undergoing gynecological surgery or examination, reported the incidence of PONV (Callesen et al., 1999; Hakim and Wahba, 2019; Massoth et al., 2021; Chen et al., 2022; Choi et al., 2022; Cha et al., 2023). The incidence of PONV was significantly lower in the OFA group than that in the control group (RR = 0.52; M-H, Fixed, 95% CI = 0.40–0.66; *p* < 0.00001; I^2^ = 12%) (Figure 3A). The heterogeneity of result was low.

Three of the 6 studies reported the use of postoperative antiemetics (Callesen et al., 1999; Massoth et al., 2021; Choi et al., 2022). The use of postoperative antiemetics was significantly lower in the intervention group than that in the control group (RR = 0.64; M-H, Fixed, 95% CI = 0.42–0.97; *p* = 0.03; I^2^ = 26%) (Figure 3B). The heterogeneity of result was moderate.

### Pain score and analgesic use

**FIGURE 4:**
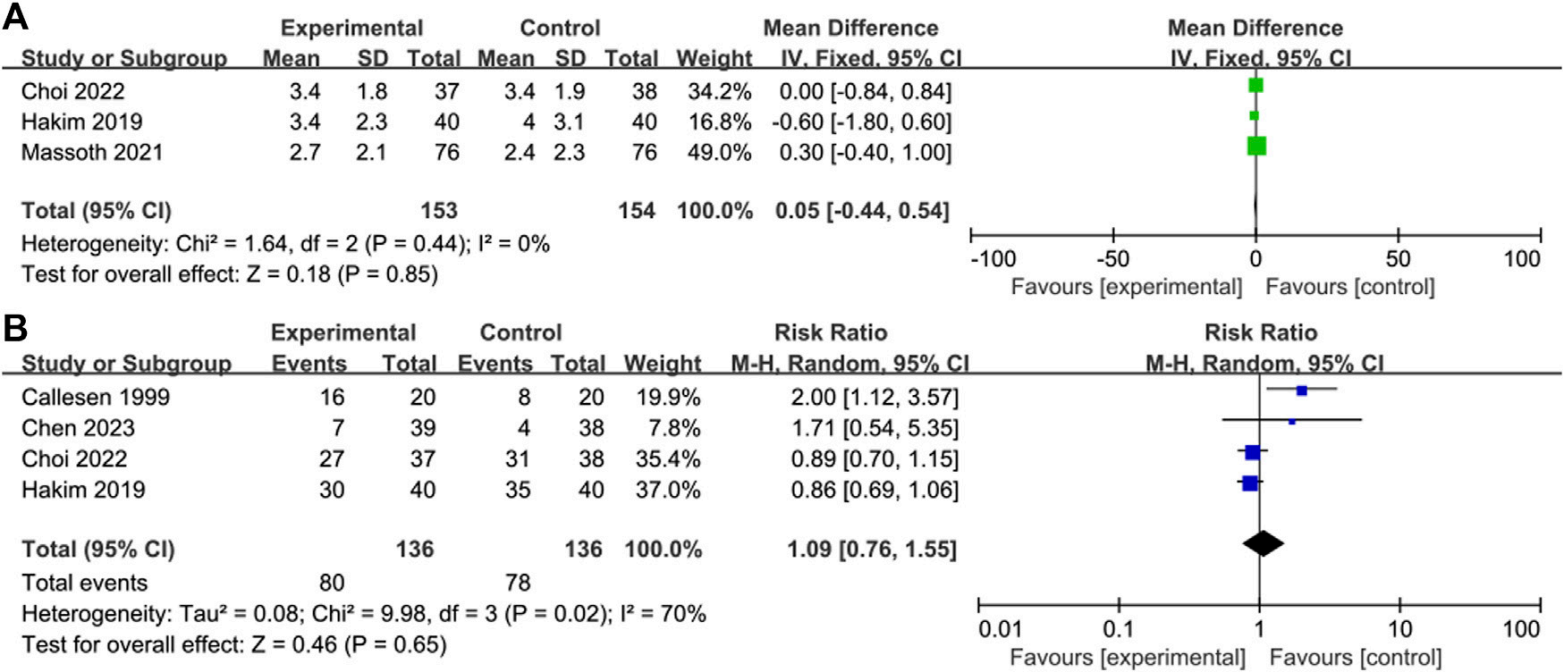
Forest plot of the **(A)** pain score **(B)** analgesic use: opioid-free anesthesia vs control.

Three of the 6 studies reported specific pain scores (Hakim and Wahba, 2019; Massoth et al., 2021; Choi et al., 2022) and there was no significant difference in postoperative pain scores between the control and OFA groups (MD = 0.05; IV, Fixed, 95% CI = −0.44–0.54; *p* = 0.85; I^2^ = 0%) (Figure 4A). The heterogeneity of result was low.

Four of the 6 studies reported the use of postoperative analgesics (Callesen et al., 1999; Hakim and Wahba, 2019; Chen et al., 2022; Choi et al., 2022); the use of postoperative analgesics in the intervention group was not significantly lower than that in the control group (RR = 1.09; M-H, Random, 95% CI = 0.76–1.55; *p* = 0.65; I^2^ = 70%) (Figure 4B). The heterogeneity of result was high.

### Time of extubation and QoR-40

**FIGURE 5:**
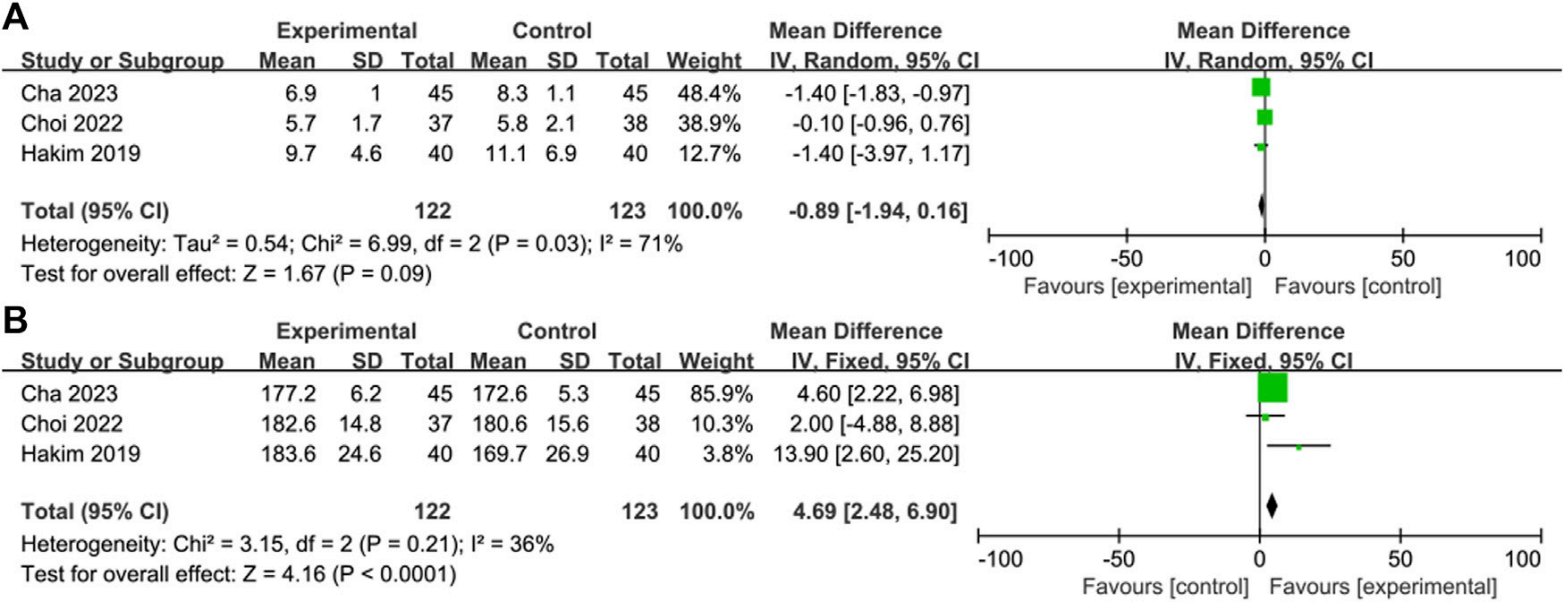
Forest plot of the **(A)** time of extubation: opioid-free anesthesia vs control **(B)** the QoR-40: opioid-free anesthesia vs control.

Three of the 6 studies reported the time of extubation (Hakim and Wahba, 2019; Choi et al., 2022; Cha et al., 2023); the time of extubation in the OFA group was not significantly shorter than that in the control group (MD = −0.89; IV, Random, 95% CI = −1.94–0.16; *p* = 0.09; I^2^ = 71%) (Figure 5A). The heterogeneity of result was high.

Three of the 6 studies reported QoR-40 scores (Hakim and Wahba, 2019; Choi et al., 2022; Cha et al., 2023). The score of QoR-40 in the OFA was significantly higher than that in the control group (MD = 4.69; IV, Fixed, 95% CI = 2.48–6.90; *p* < 0.0001; I^2^ = 36%) (Figure 5B). The heterogeneity of result was moderate.

### Subgroup analysis

**FIGURE 6:**
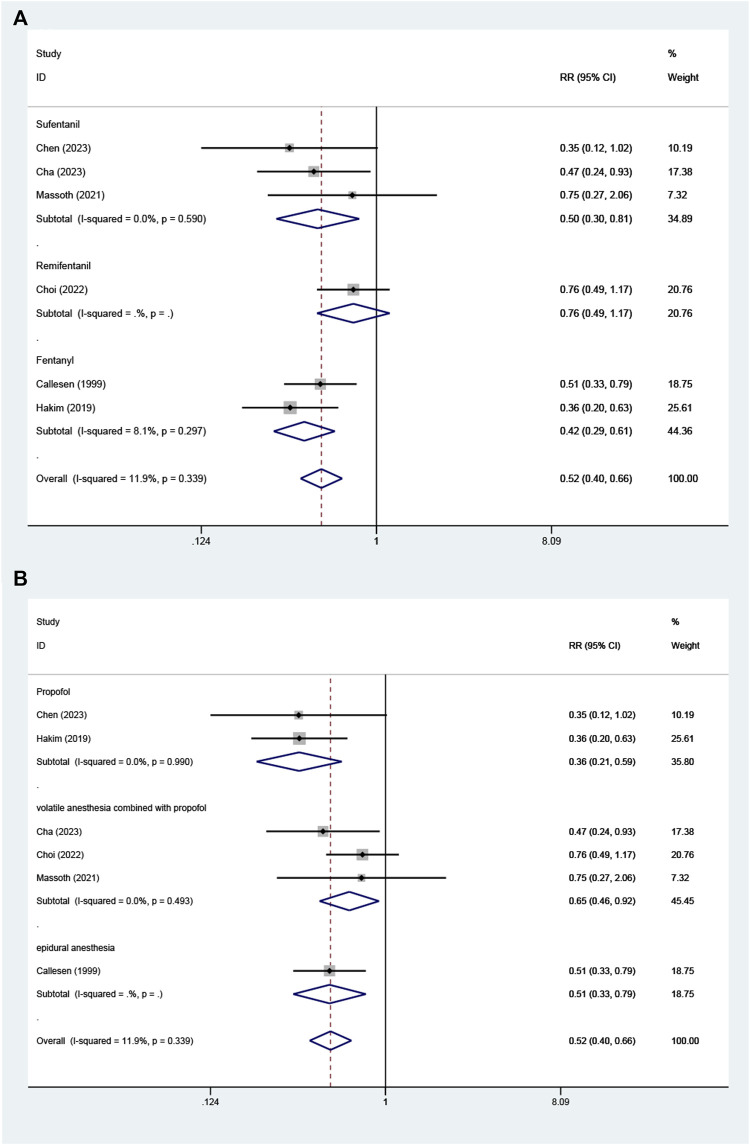
Subgroup analysis. Forest plot of the **(A)** according to type of intraoperative opioid use (remifentanil vs sufentanil, fentanyl) **(B)** according to methods used for maintenance of opioid-free anesthesia (volatile anesthesia combined with propofol vs propofol vs epidural anesthesia).

**FIGURE 7:**
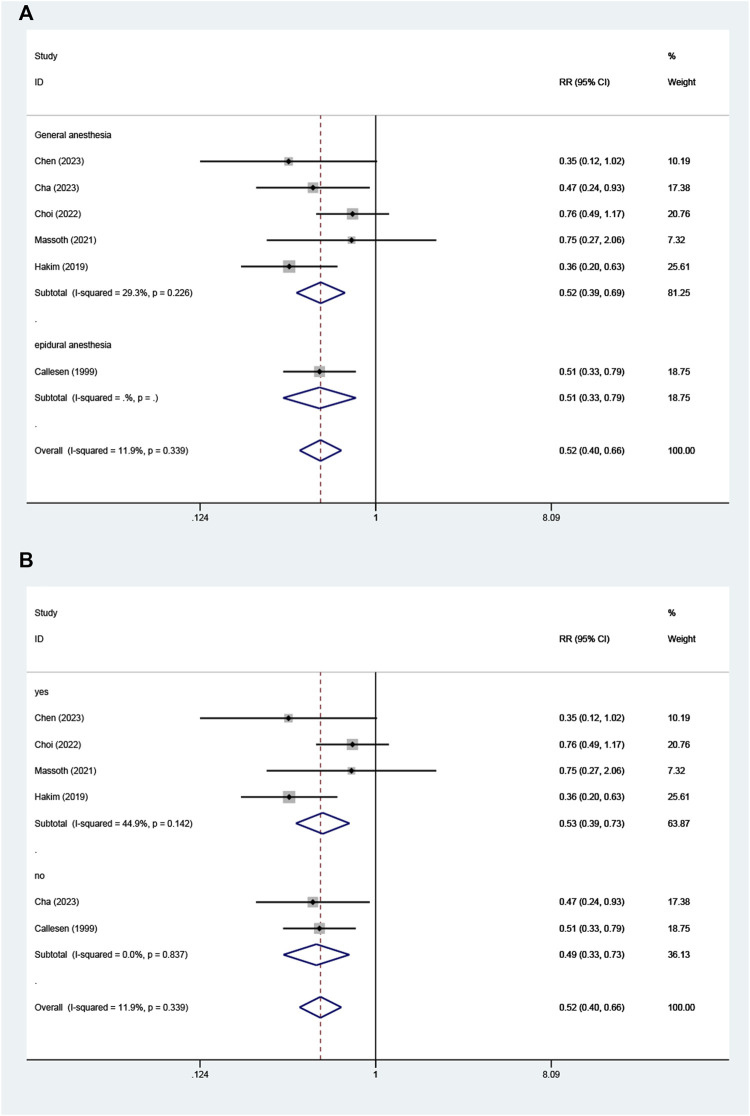
Subgroup analysis. Forest plot of the **(A)** according to methods of implementation of opioid-free anesthesia (epidural vs general anesthesia) **(B)** according to whether dexmedetomidine is used in opioids anesthesia (yes vs no).

Subgroup analysis of intraoperative opioid use showed that the heterogeneity of each subgroup was reduced (Figure 6A). When it based on intraoperative maintenance of opioid-free anesthesia also showed a decrease in heterogeneity among subgroups (Figure 6B). However, subgroup analysis according to methods of implementation of opioid-free anesthesia did not show a reduction in heterogeneity across subgroups (Figure 7A), nor did subgroup analysis according to whether dexmedetomidine was used in the opioid-free anesthesia group (Figure 7B).

### Sensitivity analysis

**FIGURE 8:**
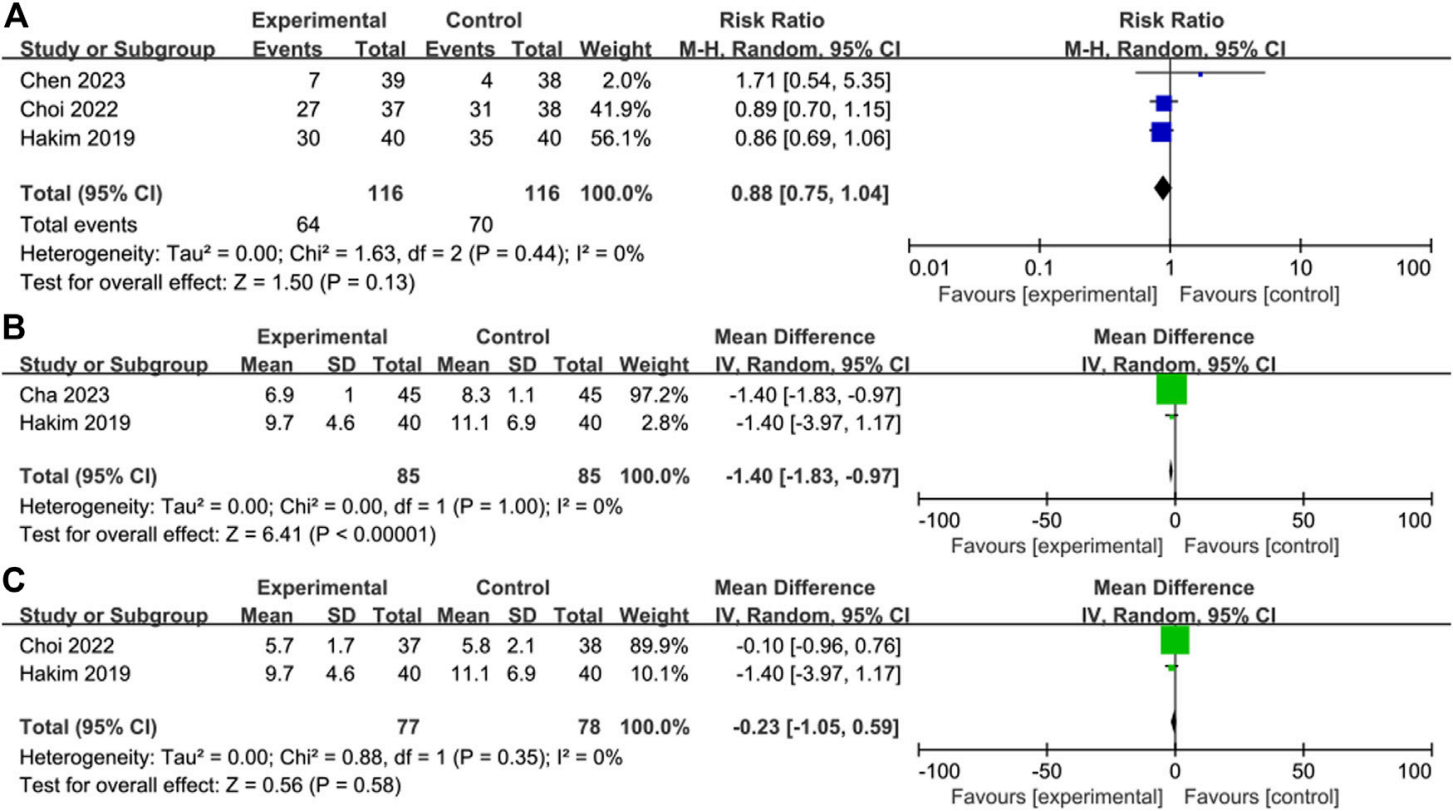
Sensitivity analysis. Forest plot comparing the **(A)** analgesic use without Callesen’s report **(B)** time of extubation without Choi’s report **(C)** time of extubation without Cha’s report.

For the outcome of postoperative analgesic use, when the study by Callesen et al. (Callesen et al., 1999)was removed, the heterogeneity of the meta-analysis was reduced; however, the conclusion keeps consistent (I^2^ = 0%; *p* = 0.13) (Figure 8A). For time of extubation, when the study by Choi et al. (Choi et al., 2022) was removed, the heterogeneity decreased; however, the conclusion did change (I^2^ = 0%; *p* < 0.00001) (Figure 8B). When the study by Cha et al. (Cha et al., 2023) was removed, the heterogeneity was also reduced, but the conclusion did not change (I^2^ = 0%; *p* = 0.58) (Figure 8C).

## Discussion

Although many previous studies have investigated the postoperative outcomes of OFA and opioid-based anesthesia, there is a lack of systematic reviews and meta-analyses addressing a particular type of surgery. In this meta-analysis, we defined opioid-free anesthesia as the absence of opioid use during surgery, and regional anesthesia was also defined as a technique of opioid-free anesthesia, which was ultimately included in six studies. The results revealed that, in gynecological surgery, compared with opioid-based anesthesia, OFA reduced the incidence of PONV and the use of postoperative antiemetics, improved the quality of recovery but did not affect postoperative pain and the time of extubation.

Previous studies have found that opioid-free anesthesia can reduce postoperative nausea and vomiting in patients (Frauenknecht et al., 2019; Salomé et al., 2021; Feenstra et al., 2023; Malo-Manso et al., 2023; Zhang et al., 2023) and improve the quality of postoperative recovery (Feenstra et al., 2023), which is consistent with the results of this meta-analysis of opioid-free gynecological surgery. But two meta-analyses found that opioid-free anesthesia reduced postoperative pain in patients (Malo-Manso et al., 2023; Zhang et al., 2023) and one showed that opioid-free anesthesia reduced extubation time (Zhang et al., 2023), which is different from our results.

PONV remains a major challenge. Female sex is an independent risk factor for PONV, and the incidence of PONV in gynecological surgery is high among various types of surgery (Apfel et al., 2012). A study by Apfel et al. reported that the incidence of PONV can reach 80% in gynecological surgery involving opioid-based anesthesia (Madej and Simpson, 1986; Wesmiller et al., 2017). Opioids are commonly used perioperative analgesics with good analgesic effect; however, they also have adverse reactions such as nausea and vomiting (Gustafsson et al., 2023). Opioids may induce nausea and vomiting by direct action on chemo-trigger zone receptors in the brainstem (Andrews, 1992). The effect of perioperative opioid use on PONV has been extensively studied, and there is strong evidence supporting that the incidence and severity of PONV are dose-dependent with perioperative opioids. (Roberts et al., 2005). OFA replaces opioids with other drugs or anesthetic techniques during anesthesia, thus minimizing perioperative opioid use, thus may reduce the incidence of PONV, which is consistent with the conclusions of this meta-analysis. Among the 6 included studies, Callesen et al. used combined spinal-epidural anesthesia in the OFA group, and regional anesthesia may have a different risk of PONV compared with general anesthesia, which may affect the accuracy of the results. (Liu et al., 2005). However, when we excluded this study, the conclusions did not change. And through subgroup analysis, we found that the sources of heterogeneity were differences in intraoperative opioid use and methods of intraoperative anesthesia maintenance. Of the included studies, 4 selected dexmedetomidine as part of OFA, and previous studies have shown that dexmedetomidine, as an α2 receptor agonist, can reduce the occurrence of PONV by modulating the release of 5-hydroxytryptamine and dopamine (Zhao et al., 2023). Despite inconsistent combinations of anesthetic drugs, we concluded that OFA is beneficial in reducing PONV during gynecological surgery.

The effect of OFA on postoperative pain is controversial; in this meta-analysis, there were no statistical differences in postoperative pain scores and postoperative analgesic consumption between the two groups. Our results support the use of multimodal analgesia to minimize opioids use and achieve adequate analgesia. However, due to differences in postoperative analgesia and multi-modal analgesia protocols, the confidence of this conclusion is limited, and more trials are needed to explore the relationship between OFA and postoperative pain. There was no difference in extubation time between the two groups. It is generally expected that opioid use will enhance sedation and thus prolong extubation time, but this result is contrary. This may be due to the fact that the insufficient sample size makes this conclusion less reliable. In addition, this meta-analysis concluded that OFA improved the quality of recovery. The included studies selected the QoR-40 as the assessment tool for quality of recovery, and PONV is an important component of QoR-40; thus, a lower incidence of PONV may lead to a better postoperative quality of recovery. However, due to the small number of included studies and high heterogeneity, the confidence of secondary outcomes is limited.

This meta-analysis had some limitations. First, we included only 6 studies, and the sample size was small. Second, the types of procedures included in the study were not broad enough. Third, the end point of our outcome indicators was the last time period, and the relevant outcome indicators occurring in the middle time period were not extracted and analyzed; thus, confidence of secondary outcomes was limited.

## Conclusion

OFA reduces PONV and the use of antiemetic drugs. In addition, it improves the quality of postoperative recovery. However, OFA can not reduce the postoperative pain scores, analgesic use and the time of extubation. Due to the strength of the evidence, we cannot support OFA as an ideal anesthesia method in gynecological surgery, and the implementation of anesthesia strategies should be case-by-case.

## Data Availability

The original contributions presented in the study are included in the article/Supplementary material, further inquiries can be directed to the corresponding authors.
